# Social Capital as Protection Against the Mental Health Impact of the COVID-19 Pandemic

**DOI:** 10.3389/fsoc.2022.728541

**Published:** 2022-04-19

**Authors:** Erik Snel, Godfried Engbersen, Jan de Boom, Marianne van Bochove

**Affiliations:** ^1^Department of Public Administration and Sociology (DPAS), Erasmus University Rotterdam, Rotterdam, Netherlands; ^2^The Hague University of Applied Sciences, The Hague, Netherlands

**Keywords:** COVID-19, social capital, mental wellbeing, social support, trust

## Abstract

The corona pandemic has a huge impact on the mental wellbeing of the Dutch population. Based on a large-scale panel survey (*N* = 22,696) on the social impact of COVID-19, this article firstly examines which social groups are most susceptible to the mental health consequences of the pandemic. Secondly, we examine whether social capital provides protection against this impact. We find that the mental health impact of COVID-19 is considerable and that it increased over the course of 2020. Women, young people, respondents with low incomes and/or poor self-perceived health, experience relatively more fear and stress due to the pandemic. We do not find a difference between respondents with or without a migration background. Social capital (received support, trust in people and in institutions) has the expected effect: the more support and trust, the less fear and stress. There is a mediation effect. Older people, respondents with high incomes and/or good health experience less fear and stress, partly because they have more social capital. This is different for females. They would experience even more fear and stress, compared to men, were it not for the fact that they have more social capital. Hence we conclude that social capital indeed provides some protection against the negative mental health consequences of COVID-19.

## Introduction

Several studies showed that the COVID-19 pandemic has a major impact on the mental wellbeing of specific segments of the population. A survey among Dutch university students, for example, showed that over half of the respondents had trouble concentrating, felt lonely, and feared that the pandemic may adversely affect their academic progress (Caring Universities, 2020). The pandemic also led to mental health issues among nursing and care home residents, particularly when these homes were closed to visitors to prevent infections among residents. Many residents experienced anxiety, sombreness, and loneliness. While nursing and care homes were closed to protect older people in need of care, this actually resulted in harmful mental health effects, perhaps even increased the mortality risks (Van der Roest et al., 2020).

These early Dutch studies are in line with the findings of the first international studies on these issues, which found that the COVID-19 pandemic had moderate, in some cases severe consequences for mental health: feelings of depression, anxiety, loneliness, etcetera (Holmes et al., 2020; Kim and Laurence, 2020; Pierce et al., 2020; Sønderskov et al., 2020; Wang et al., 2020; Xiong et al., 2020; Zhang and Ma, 2020). Based on a survey among American adults, Kim and Laurence (2020, p. 710) conclude that pre-existing inequalities in health status and job stability amplify the negative mental health consequences of COVID-19-induced restrictions. Their findings also suggest that the mental impact of restrictive measures increased over time. A first Dutch study, however, found only limited mental health consequences of COVID-19 during the first months of the pandemic. Feelings of emotional loneliness were up only slightly after the outbreak of the virus, while symptoms of anxiety and depression actually decreased compared to pre-Corona times (Van der Velden et al., 2021). Our study comes to different outcomes, possibly because it spans a longer period of time, during which—as we shall see—the mental health consequences of the pandemic intensified. The purpose of our study is to determine which groups of the Dutch population (particularly in terms of social class and ethnicity) were affected most by the pandemic's adverse mental health consequences. Furthermore, we will study whether having social capital offers some level of protection against pandemic-related feelings of anxiety and stress.

This study is based on a large-scale survey about the societal impacts of COVID-19 conducted in the Netherlands in November 2020 (*N* = 22,696). Compared to two previous surveys held in April 2020 and July 2020, this November survey showed a strong increase in mental distress (cf. Engbersen et al., 2020). It is important to note that these three survey waves were conducted in different contexts. The corona virus hit the Netherlands in several waves. After the first wave of infections in March and April 2020 came the summer months when the virus seemed to slowly disappear from the Netherlands. However, in the fall of 2020, the virus started to spread again, ushering in the predicted second wave. As early as in April 2020, it was clear that one quarter of our respondents more often felt anxious, nervous, irritable, and stressed since the outbreak of COVID-19. In July 2020, these feelings of pandemic-related anxiety and stress eased, but they quickly soared again in November 2020, when colder weather conditions accelerated the spread of the virus and the Dutch government started to impose strict measures to curb the virus. Particularly striking were the feelings of hopelessness due to the COVID-19 pandemic. In November, nearly 40% of respondents felt they “have nothing to look forward to” (Engbersen et al., 2020, p. 10).

This contribution takes a sociological perspective to examine the extent to which the Dutch population experiences feelings of anxiety and stress because of the pandemic and the measures taken in response. We will do this in two ways. First, we examine which social groups experience such feelings of anxiety and stress and to what degree. Our second and central focus is whether social capital, i.e., people's social contacts and networks and their level of trust in “people in general” (“general” or “social trust”) and in institutions (“institutional trust”), offers protection against the mental health consequences of the pandemic. We expect this protective effect, because social contacts work as a buffer against everyday anxieties, and provide support and relevant health information. Trust in other people promotes mutual collaboration. People with institutional trust will be more compliant to the regulations by the authorities during the pandemic. The article is structured as follows. In the next section, we discuss existing literature on the correlation between social class, social capital, and (mental) health differences, with a specific focus on recent research on the effects of the COVID-19 crisis. After explaining our research method, we describe our findings with respect to (1) which social groups are hit harder by the adverse effects of the pandemic and, (2) whether social capital provides protection against these effects. We conclude with a discussion of the theoretical implications of our findings.

## Literature Review: Social Class, Social Capital, and the Mental Health Consequences of COVID-19

### The Role of Social Class and Migration Background

Socioeconomic health differences in the Netherlands are a well-documented fact. Less-educated people in the Netherlands live, on average, 6 years less than people with higher educational qualifications. The differences in life expectancy in good health are even greater. The less-educated have 15 fewer years of healthy life expectancy than the highly educated (cf. Mackenbach, 2012; Mackenbach et al., 2016)^1^.

The question is whether we can see similar socio-economic health differences in the case of COVID-19. Although it was said initially that “corona does not discriminate”, both international and Dutch research showed social and ethnic disparities in the extent to which people are affected by the virus (Hawkins et al., 2020; Millet et al., 2020; Patel et al., 2020; Public Health England, 2020). A study by Netherlands Statistics showed that the mortality rate due to COVID-19 during the first months after the virus outbreak was twice as high for the lowest income quintile in the Netherlands compared to the highest quintile. Ethnic differences in mortality rates due to COVID-19 were smaller also because the pandemic began and caused many deaths in a Dutch region with relatively few non-Western migrants. However, in the Dutch major cities where many non-Western immigrants live, the mortality rate due to COVID-19 for persons with a non-Western migration background was 50% higher than for native residents of these cities (De Visser et al., 2021). The latter findings confirm previous research which found an overrepresentation of individuals with a non-Western migration background in the number of hospital admissions due to COVID-19 in Amsterdam (Coyer et al., 2021).

There are various reasons why both low-income groups and minority groups are hit harder by COVID-19. Both lower educated natives and non-Western migrants are more likely to have the well-known risk factors (obesity, diabetes, etc.) that also play a role in COVID-19 infections. In addition, people from both groups are more likely to have jobs involving a higher risk of infection—e.g., construction, cleaning, personal services—and offering fewer options for home working, which also increases the chance of infection. Besides, they often live in poorer houses with less space, which may promote the spread of the virus. Both low educated (or illiterate) natives and migrants with insufficient language command may also have restricted access to adequate health information and to health care (Patel et al., 2020; Cockerman, 2021). Finally, some cultural factors that may play a role. For instance, a stronger bond with their own community and extended family may increase the risk of infection at family gatherings.

This study does not contain data about the number of COVID-19 infections or mortality rates. We examine the impact of the pandemic on people's mental wellbeing as one of the aspects of the social impact of COVID-19. More in particular, our focus is on whether the mental health consequences of the COVID-19 pandemic differ by social status (educational attainment and income level) and by migration background. Based on existing research, we expect a positive relationship between respondents' social status and their subjective wellbeing. People with a lower level of education and with lower income are more often confronted with financial strain, while lacking knowledge and coping strategies to adequately handle such strain, resulting in mental tension and insecurity (Holmes et al., 2020; Kim and Laurence, 2020; Pierce et al., 2020; Wang et al., 2020; Xiong et al., 2020).

Research about the mental health consequences of the COVID-19 pandemic also systematically point to low levels of income and education as risk factors, although the positive effect on wellbeing diminishes gradually for the highest incomes (Holmes et al., 2020; Kim and Laurence, 2020; Pierce et al., 2020). One may also question the direction of this correlation. Does lower educational attainment and income lead to greater strain and less wellbeing? Or are persons with these kinds of feelings less able to reach higher levels of education and acquire a higher income? We expect to see stronger feelings of anxiety and stress among respondents with lower educational and income levels, if only because they are more likely to work jobs that involve a greater risk of infection, are less able to work from home, or tend to have less job security.

We also expect persons with a non-Western migration background to experience higher levels of anxiety and stress because of the COVID-19 crisis. We already mentioned various economic and possible cultural factors that cause people with a non-Western migration background to be more exposed to the risk of infection, which can lead to more anxiety and stress. In fact, research by Stronks et al. (2020) showed that the chance of depression is twice or even three times as high among Dutch people with Surinamese, Turkish, and Moroccan background as it is among Dutch people without a migration background, although these differences are partly due to differences in social status between the various population groups.

### The Role of Social Capital

Our second question is whether social contacts, social networks and trust, grouped together under the term “social capital”, offer protection against the pandemic's adverse mental health consequences.

Although scholars (Bourdieu, 1986; Putnam, 2000) give different definitions of social capital, they generally agree on three core aspects of social capital: social and institutional trust, norms of reciprocity and social networks. Sociologists also show that social capital benefits both people's individual life opportunities and the functioning of social communities in many ways; our study concentrates on the correlation between social capital and people's mental health (Coleman, 1990; Portes, 1998).

Empirical research generally showed that social capital results in positive health outcomes, although this differs with the various forms and levels of social capital and the examined health outcomes (see for overviews: De Silva et al., 2005; Song et al., 2016; Cockerman, 2021). Social contacts and networks give access to social support and valuable health information. Social contacts and networks also work as a protective buffer against everyday tensions and anxieties. People with many social contacts experience negative events such as the pandemic as less threatening or have less of an emotional response to them (Putnam, 2000, p. 332; Kawachi and Berkman, 2001). Social capital also implies social norms, such as maintenance of healthy norms and promotion of health behaviour (Nieminen et al., 2013). Other studies showed that the trust dimension of social capital contributes to people's subjective experience of wellbeing (Portela et al., 2013), self-reported mental health (Lindström, 2008) and reduces major mental depressions (Fujiwara and Kawachi, 2008). People with high levels of social and institutional trust will engage more in solidaric practices and will be more compliant to hygienic and preventive behaviour required by the authorities (Makridis and Wu, 2021).

High levels of social capital also enhance the ability of individuals and communities to cope with crises (Makridis and Wu, 2021). Drawing on two American studies, Tierney (2019) claimed that social capital contributes to people's resilience, making them better able to withstand the adverse health effects of natural disasters such as extreme weather, flooding, and epidemics. First, Klinenberg (2002) showed that the extreme heat wave in Chicago in July 1995 resulted in higher mortality rates in a poor and isolated Chicago neighbourhood than in a qua social status comparable district with many shops and restaurants, therefore more people in the streets, more social contacts and, in general, more community life. Social capital, Klinenberg argued, protects otherwise vulnerable residents, which explains the lower mortality rate in this neighbourhood.

Secondly, Adeola and Picou (2014) studied mental health symptoms [depression, post-traumatic stress disorder (PTSD)] among Katrina survivors 3 years after the hurricane. They found that mental health symptoms were still widespread, but that they were more prevalent in certain population groups (African Americans, women, single adults, and people with weaker social networks). Lack of social capital was the strongest predictor of long-term health problems. Adeola and Picou (2014) explained this by the positive influence of networks: social contacts can both set an example of healthy lifestyles and work like a buffer. People with many social contacts experience negative events as less threatening or have less of an emotional response to them.

The positive effects of social capital are also shown in recent studies on the Corona pandemic. Although social interactions foster infections, research shows that social capital is in fact negatively associated with COVID-19 growth rate and with COVD-19 deaths and hospitalizations (Varshney and Socher, 2020; Borgonovi et al., 2021; Makridis and Wu, 2021). There are various explanations for this association. Social networks provide people with health-related information, which in turn may result in health protective behaviour such as wearing face masks, washing hands frequently, and avoiding unnecessary social contacts. Individuals in communities with strong norms of reciprocity and social solidarity may be more aware of the psychological costs if they infect others. Trusting people have more concern for others, leading to more hygienic practices and social distancing. High levels of institutional trust may result in more compliance to public health recommendations such as social distancing, mask wearing and vaccination (Barrios et al., 2020; Borgonovi and Andrieu, 2020; Varshney and Socher, 2020; Borgonovi et al., 2021; Ferwana and Varshney, 2021; Makridis and Wu, 2021).

There is a growing body of studies on the role of social capital in reducing the mental health consequences of the pandemic with mixed outcomes. Chan et al. (2021) found that social capital has indeed a negative effect on mental health problems resulting from the pandemic, but only for the non-active population. For economically active individuals, employment and financial stability are more important than social support. They also found that social capital did not affect subjective wellbeing in times of corona. Wang et al. (2021) found that psychosocial support, in particular from the near family, lowered the negative effect of the pandemic on feelings of loneliness, but not on the mental health of respondents. Paolini et al. (2020) found that social and political trust had a significant positive effect on the subjective wellbeing of Italians in the early days of the pandemic, but not on the level of distress of respondents. Van Tilburg et al. (2021), on the other hand, found in a study on Dutch elderly that a decline in institutional trust was associated with increased mental health problems and emotional loneliness.

In our analysis, we distinguish two dimensions of social capital, each with two different indicators: the relational or network dimension (social contacts as such and social support exchanged within social networks) and the cognitive or trust dimension (general trust and institutional trust). We analyse the impact of these different dimensions and indicators of social capital separately. The ratio for doing so, besides methodological reasons^2^, lies in previous research which showed that different forms of social capital produce different social outcomes. Yip et al. (2007), in a study in rural China, found that trust is positively associated with various health outcomes (self-reported health, psychological health, subjective wellbeing), but that there is “little statistical association” between organizational membership (as indicator of the network dimension of social capital) and these outcomes. Similarly, Ding et al. (2020) found that two forms of social capital—community engagement and individual commitment to social institutions—have opposite impacts on social distancing during the corona pandemic. The first is measured by membership of clubs and associations (which relates to the network dimension of social capital), the latter is measured by voting and contributing to social institutions (related to the trust dimension of social capital). And Makridis and Wu, as already mentioned, concluded that “..social capital affects response to COVID-19 through trust and norms, rather (than) social networks and trust” (Makridis and Wu, 2021, p. 14).

Based on these insights, we expect that both dimensions of social capital (networks and trust) provide a certain level of protection against the adverse mental health consequences of the COVID-19 crisis.

### Hypotheses

We derive four hypotheses from our overview of relevant studies on social class, social capital and mental health consequences:

- Hypothesis 1: People with a lower social status in terms of education and income experience more anxiety and stress as a result of the COVID-19 pandemic;
- Hypothesis 2: People with a non-Western migration background experience more anxiety and stress as a result of the COVID-19 pandemic;
- Hypothesis 3: People with more social capital in terms of social networks and social support experience less anxiety and stress as a result of the COVID-19 pandemic;
- Hypothesis 4: People with more social capital in terms of institutional and general trust experience less anxiety and stress as a result of the COVID-19 pandemic.

## Data and Methods

### Data, Sample, and Weighting

The data used in this study are derived from a large-scale online survey on the social impact of the COVID-19 pandemic in the Netherlands conducted in November 2020 by election research institute Kieskompas (Engbersen et al., 2020).

For data collection, Kieskompas used their national panel, which is a representative stratified sample (stratified random sampling) of the Dutch voting population (18+ years). The questionnaire was distributed among 48,329 Kieskompas panel members, 19,577 panel members returned the questionnaire (40.5% response rate). Additionally, three cities participating in the research (Amsterdam, The Hague, and Rotterdam) employed extra activities to include more underrepresented groups. They placed adds on Facebook, disseminated an anonymous participation link for the survey in a targeted manner, one city distributed the questionnaire among their own city panel. In the end of the fieldwork period, the questionnaire could be filled in through an anonymous participation link. These activities resulted in about 5,500 extra respondents.

In order to make the results generalizable for the Dutch voting population, Kieskompas applied a weighting to make the survey results representative for education, age, gender, region, migration background and voting behaviour. Questionnaires with insufficient information about these characteristics were excluded from the sample. All of this resulted in a sample of 22,696 respondents which is representative for the (adult) Dutch population as a whole.

### Measurements

The dependent variable in our study is a scale describing the level of anxiety and stress experienced due to the COVID-19 pandemic. This scale is based on respondents' responses to six statements in the questionnaire, the first of these being: “Ever since the COVID-19 outbreak in the Netherlands, I more often feel anxious.” In the same way, respondents were asked whether they, since the COVID-19 outbreak, more often felt nervous, stressed or irritable, found it harder to relax, and if they felt like there was nothing to look forward to. These questions are based on the *Depression Anxiety Stress Scale* (DASS) [earlier translated to Dutch by De Beurs et al. (2001)]. We asked respondents to (strongly) disagree or (strongly) agree with these statements (1–5). Their answers were plotted on an “anxiety and stress” scale with the average score on the six items. The scale turned out to be very reliable (Cronbach's Alpha 0.89).

Respondents' migration background, socio-economic status, and social capital are the explanatory variables in the study. Respondents' *migration background* was determined based on their country of birth and both their parents' country of birth and recoded into three categories: Dutch people without a migration background (reference category), Dutch people with a Western migration background, and Dutch people with a non-Western migration background. The level of education and net monthly income are indicators of someone's socio-economic status. Respondents' *level of education* was measured using three dummy categories: low (no education, only primary education up to level 1 secondary vocational education; the reference category), medium (second-stage secondary or pre-university education, levels 2–4 secondary vocational education), and high (higher professional education, university education, bachelor and higher). Respondents' *net monthly income* was classified in five dummy categories: minimum income (under €1,150 for single-person households and €1,600 for multiple-person households; reference category), between minimum and modal income (from €1,150 for single-person households/€1,600 for multiple-person households to €2,150), modal to double modal income (€2,150–€3,500), between double and three times modal income (€3,500–€5,000), and over three times modal income (€5,000 or more).

Respondents' social capital was established using four indicators: social contacts, social support received or expected, general trust, and institutional trust. Three indicators were captured as scales on which multiple responses (items) are combined. The *social contact scale* is based on a question about social encounters, contacts by phone and/or through written communication, and online contacts with people with whom the respondent does not share a household. Respondents were asked about the frequency of their contacts with family members, friends or close acquaintances, neighbours, or other people in their local community. The possible answers ranged from “almost daily,” “at least once a week,” “two or three times a month,” and “once a month,” to “under once a month” and “never”. The social contact scale is the average score on these four items. A high score on the contact scale means frequent contact, while a low score means little to no contact. This scale turned out to be moderately reliable (Cronbach's Alpha 0.664).

The *help received scale* is based on the question: “If you were to need help personally due to the COVID-19 outbreak, who from outside your household would you expect to be there for you?” Respondents were asked to indicate for family members who live elsewhere, friends, neighbours, or acquaintances (four items) whether they were “already receiving help from them” or would “definitely,” “maybe,” or “certainly not” expect help from them if needed. This scale also turned out to be moderately reliable (Cronbach's Alpha 0.597). The *institutional trust scale* measures the level of trust in the national government, local government, Netherlands Institute of Public Health and Environmental Protection (Dutch acronym: RIVM), and municipal health service (Dutch acronym: GGD). In response to questions about their level of trust in these institutions, respondents had a choice between whether they trust these institutions very little, little, not much/not little, do trust them, or have a lot of trust in them (1–5). The institutional trust scale showing the average score on these four items was highly reliable (Cronbach's alpha 0.858). The *trust in people* (or *general trust*) scale is based on a single question that asked respondents to indicate how much they trust “people in general” (1–5).

Finally, several control variables were included in the analyses. *Sex* was included with “male” as the reference category. *Age* was included as a continuous variable. *Home situation*, originally with six categories, was recoded and included in two dummy categories: single-person households (reference category) and multi-person households. *Perceived health*, measured in five categories, was recoded into four categories: (very) poor (reference category), moderate, good, and very good.

The descriptive information on the variables is listed in Table 1. Missing data was deleted using the listwise deletion method. The rate of missing data as per the listwise deletion method ranges from 0% (age) to 8.5% (income). The loss of respondents due to listwise deletion is relatively high, due mainly to the missing answers to the question about respondents' income. Associations between the variables are displayed in Table 2. Analyses demonstrated that most variables were significantly correlated with each other in our sample, however most of them are not substantial (<0.4).

**Table 1:** Descriptive statistics.

	**Mean**	**S.d**.	**Min**.	**Max**.
Anxiety and stress (April 2020)	2.49	0.96	1.00	5.00
Anxiety and stress (July 2020)	2.44	0.89	1.00	5.00
Anxiety and stress (November 2020)	2.67	0.94	1.00	5.00
**Gender**				
Male	0.50		0.00	1.00
Female	0.50		0.00	1.00
*Age (in years)*	49.97	17.59	18.00	96.00
**Migration background**				
Native	0.81		0.00	1.00
Western	0.10		0.00	1.00
Non-Western	0.08		0.00	1.00
**Living situation**				
Oneperson household	0.24		0.00	1.00
Morepersons household	0.76		0.00	1.00
**Health**				
(Very) bad	0.04		0.00	1.00
Moderate	0.19		0.00	1.00
Good	0.58		0.00	1.00
Very good	0.17		0.00	1.00
**Education**				
Low	0.25		0.00	1.00
Mediate	0.43		0.00	1.00
High	0.32		0.00	1.00
**Income**				
Minimum	0.14		0.00	1.00
Minimum to modal	0.21		0.00	1.00
Modal to 2x modal	0.29		0.00	1.00
2x modal to 3x modal	0.24		0.00	1.00
More than 3x modal	0.12		0.00	1.00
*Social contacts (0–5)*	2.77	1.05	0.00	5.00
*Support received (1–4)*	2.35	0.51	1.00	4.00
*Trust in people in general (1–5)*	3.23	0.94	1.00	5.00
*Trust in institutions (1–5)*	3.39	1.00	1.00	5.00

**Table 2:** Correlation of all variables in the analyses (unweighted).

			**Migration background**											
	**Gender**	**Age**	**No migration background**	**Western background**	**Non-western background**	**Living situation**	**Eduation**	**Income**	**Health**	**Social contacts**	**Support received**	**Trust in people in general**	**Trust in institutions**	**Anxiety and stress**
Gender	1													
Age	−0.089**	1												
No migration background	−0.28**	0.018**	1											
Western background	0.022**	0.038**	−0.867**	1										
Non-western background	0.016*	−0.104**	−0.448**	−0.058**	1									
Living situation	−0.080**	−0.096**	0.033**	−0.029**	−0.014*	1								
Eduation	0.056**	−0.139**	−0.059**	0.054**	0.020**	0.017*	1							
Income	−0.122**	−0.070**	0.005	0.002	−0.012	0.421**	0.289**	1						
Health	−0.026**	−0.184**	0.001	−0.012	0.019**	0.106**	0.156**	0.195**	1					
Social contacts	0.065**	0.072**	0.001	0.014*	−0.028**	0.041**	0.050**	0.076**	0.055**	1				
Support received	0.041**	0.154**	0.016*	−0.004	−0.025**	0.020**	0.005	0.055**	0.052**	0.317**	1			
Trust in people in general	0.048**	0.107**	0.026**	−0.006	−0.042**	0.062**	0.120**	0.140**	0.146**	0.166**	0.234**	1		
Trust in institutions	−0.009	−0.068**	0.017*	−0.014*	−0.008	0.041**	0.172**	0.156**	0.169**	0.026**	0.115**	0.286**	1	
Anxiety and stress	0.100**	−0.237**	−0.009	0.000	0.017*	−0.017*	0.011	−0.080**	−0.191**	−0.068**	−0.141**	−0.157**	−0.122**	1

*For correlations of numeric variables Pearsons's r is used (marked grey), for nominal or ordinal variables Spearman's rho*.

** *Correlation is significant at the 0.01 level (2-tailed)*.

* *Correlation is significant at the 0.05 level (2-tailed)*.

### Analysis Strategy

The ultimate goal of our analysis is to examine whether social capital mediates the adverse mental impact of the COVID-19 pandemic. To do so we have to examine the mutual associations between three groups of factors (demographic characteristics, experienced anxiety and stress since the Corona outbreak, and social capital). To examine these associations, we use linear regression analysis in multiple steps^3^. First, we analyse the correlation between the demographic characteristics and anxiety and stress: who experiences the mental health impact of the pandemic? Secondly, we analyse (a) the association between the demographic characteristics and the four social capital indicators and (b) the association between the social capital indicators and anxiety and stress. Finally, we include all variables into one regression model. We assume that social capital has the expected protective effect when the initial mental health impact of the pandemic disappears or diminishes after including social capital into the analysis in the last step.

## Outcomes: The Mental Health Consequences of the COVID-19 Pandemic

Our study aimed to identify the social impact of the COVID-19 pandemic, in particular the mental health impact of the pandemic. Do respondents experience more anxiety and stress since the Corona outbreak? In our survey, conducted in November 2020, the average score was 2.7 on a scale from 1 to 5. This means that the feelings of anxiety and stress caused by the COVID-19 crisis were higher in November 2020, during the run-up to the second wave of the pandemic, than in previous stages of the pandemic, including the outbreak of the virus in April 2020 (average of 2.5) (see Table 1).

The remainder of this article will be based solely on cross sectional data from the third survey (November 2020). Appendix 1 to this article gives the outcomes of additional linear regression analyses.

### Social Class and Mental Health Consequences of COVID-19

In Table 3, we analyse the association between demographic characteristics of respondents and their experienced level of anxiety and stress (Model 1). These results enable us to examine whether certain social groups experience more anxiety and stress due to the pandemic than other groups, thereby testing the first two hypotheses of this study.

**Table 3:** Determinants of anxiety and stress resulting from COVID-19 (Linear regression).

	**Model 1**				**Model 4**				**Model 5**			
	***B***	**Sig**	**SE**	**ß**	***B***	**Sig**	**SE**	**ß**	***B***	**Sig**	**SE**	**ß**
(Constante)	3.912	***	0.048		4.559	***	0.057		*4.569*	***	0.056	
**Gender (male = ref)**												
Female	0.114	***	0.013	0.062	0.132	***	0.013	0.072	*0.130*	***	0.012	0.071
*Age (in years)*	–*0.015*	***	*0.000*	–*0.271*	−0.014	***	0.000	−0.253	–*0.014*	***	*0.000*	–*0.254*
**Migration background (non = ref)**												
Western	0.031		0.020	0.010	0.021		0.020	0.007	*0.025*		0.020	0.008
Non-Western	−0.059		0.036	−0.011	−0.079	*	0.036	−0.015	−0.073	*	0.036	−0.014
**Living situation (single = ref)**												
More persons household	0.013		0.016	0.006	0.015		0.015	0.007				
**Health [(very) bad = ref]**												
Moderate	−0.106	**	0.035	−0.044	−0.071	*	0.034	−0.030	−0.076	*	0.034	−0.032
Good	−0.440	***	0.033	−0.241	−0.369	***	0.033	−0.202	−0.375	***	0.032	−0.205
Very good	−0.758	***	0.035	−0.332	−0.667	***	0.035	−0.292	−0.676	***	0.035	−0.295
**Education (lower = ref)**												
Secondary	−0.044		0.023	−0.023	−0.026		0.023	−0.013	−0.029		0.022	−0.015
Higher	0.010		0.022	0.005	0.052	*	0.022	0.028	0.050	*	0.022	0.027
**Income (minimum = ref)**												
Minimum to modal	−0.057	*	0.026	−0.024	−0.038		0.026	−0.016	−0.040		0.025	−0.017
Modal to 2x modal	−0.119	***	0.024	−0.061	−0.089	***	0.024	−0.046	−0.083	***	0.024	−0.043
2x modal to 3x modal	−0.147	***	0.025	−0.072	−0.103	***	0.025	−0.051	−0.097	***	0.024	−0.047
More than 3x modal	−0.167	***	0.027	−0.071	−0.111	***	0.027	−0.048	−0.104	***	0.026	−0.044
*Social contacts (0–5)*					−0.006		0.006	−0.007				
*Support received (1–4)*					−0.120	***	0.014	−0.062	−0.124	***	0.013	−0.064
*Trust in people in general (1–5)*					−0.066	***	0.008	−0.062	−0.066	***	0.008	−0.062
*Trust in institutions (1–5)*					−0.085	***	0.007	−0.085	−0.085	***	0.007	−0.084
	*F* = 191.26***; *R*^2^ = 0.121; adj. *R*^2^ = 0.121				*F* = 176.42***; *R*^2^ = 0.141; adj. *R*^2^ = 0.140				*F* = 203.04***; *R*^2^ = 0.141; adj. *R*^2^ = 0.141			

*B, unstandardized regression coefficient; SE, standard error of the regression coefficient; ß, standardized regression coefficient; R^2^, explained variance; adj. R^2^, adjusted explained variance*.

* *p < 0.05*.

** *p < 0.01*.

*** *p < 0.001 (two tailed)*.

Model 1 in Table 3 shows that women and young people experience more anxiety and stress since the virus outbreak than men and older people (the statistically significant negative correlation between age and experienced level of anxiety and stress means: the older the person, the less anxiety and stress). Respondents with (very) good perceived health and those with a higher income (from modal income upwards) experience less pandemic-related anxiety and stress than respondents who rate their own health as “(very) poor” or who live on a minimum wage (both reference category). We do realise that low income and poor (perceived) health often go hand in hand.

In addition, various personal characteristics turn out not to correlate with the experienced level of anxiety and stress. For example, we did not find the expected association between the respondents' home situation and the experienced anxiety and stress. We expected this association because people who live on their own have fewer opportunities to share their concerns with others, especially when possibilities for social contact outside the home are restricted, than people who share a home with someone else (other adults and/or children). Jace and Makridis (2021) also found that being married works as a protective factor on mental health during the pandemic. This turned out not to be the case in our research. Perhaps people in multi-persons household worry about the others in their household, including their children. Another explanation may be that singles benefited from online social contacts to such an extent that it compensated for living alone.

We also do not find a statistically significant difference between people with higher and lower educational levels and the level of experienced anxiety and stress. This is surprising because, as mentioned above, we did see a difference in anxiety and stress levels between respondents with a minimum income on the one hand and those with an (above) average income on the other. A possible explanation is that lower educated respondents are less concentrated in vulnerable segments of the labour market (with many flexible jobs and fewer possibilities for home work) and, therefore, experience less anxiety and stress. To summarise, hypothesis 1, which expected respondents with lower social status (in terms of education and income) to experience more anxiety and stress as a result of the pandemic, is confirmed for lower income groups, but not for people with a lower level of education.

### Migration Background and Mental Health Consequences of COVID-19

We expected people with a non-Western migration background to experience more anxiety and stress as a result of the COVID-19 crisis than native Dutch respondents due to the fact that the incidence of infections among non-Western migrants is relatively higher. Model 1 (Table 3) does not confirm the expected difference. Initial bivariate analyses (not shown) established that both Western and non-Western migrants experience more anxiety and stress caused by the COVID-19 pandemic than respondents without a migration background. However, these differences disappeared after including other personal characteristics, especially education and income, in the multivariate analysis^4^. This leads to a nuanced answer to hypothesis 2. Initial differences between respondents with and without a migration background disappear when the analysis takes account of differences in social status. It is, therefore, more about people's socio-economic position than about ethnic or cultural characteristics.

What initially surprised us was that young people experience higher levels of anxiety and stress due to the COVID-19 crisis than older people, while the latter group is exposed to considerably greater health risks (see also Nearchou et al., 2020). There are various reasons why COVID-19 and the restrictions to stem its spread have major mental health consequences precisely among young people. Young people want to discover the world, meet new people, and strike up relationships. The loss of social contacts is more consequential for them than it is for older people. In addition, young people may be more susceptible to stress and depression than older age groups (Hammen, 2015). Finally, many young people may also be depressed by have low jobs, income security and mounting student debt.

### Social Capital and Mental Health Consequences

Our second research question is whether having social capital protects against the adverse mental health consequences of the COVID-19 pandemic. In other words: do respondents with higher scores on the four social capital indicators experience less pandemic-related anxiety and stress? And if so, which groups display these protective effects of social capital? To examine these issues, we conducted additional linear regression analyses. First, we examined the correlation between relevant personal features and the four social capital indicators (Table A1, Model 2a to 2d, see Appendix 1) and the association between these social capital indicators and the experienced levels of anxiety and stress (Table A1, Model 3, see Appendix 1). Finally we included all variables into the regression model (Model 4 and Model 5 of Table 3).

Model 2a to 2d (Table A1, Appendix 1) show whether the various groups among our respondents returned different scores on the various social capital indicators. Women generally score higher on most social capital indicators than men. Only in terms of institutional trust, there is no difference between the sexes. With the same three indicators, we found a positive correlation with age: the older the respondents, the higher the score. Young respondents only score higher than older people on institutional trust. When it comes to respondents' migration background, we see that respondents with a non-Western migration background return significantly lower scores on social contacts and general trust than the native-Dutch reference group. Respondents with a Western migration background also return lower scores on both general and institutional trust. Other than that, however, there is little difference between respondents with and without a migration background in their scores on the social capital indicators.

What does matter, however, is the respondents' perceived health and social position. Respondents who perceive their health as (very) good return higher scores on all four social capital indicators than those with (very) poor self-rated health, which confirms that perceived health and social capital are clearly related. In terms of educational attainment, we see that respondents with the highest level of education in particular return significantly higher scores on nearly all the social capital indicators than the reference group of lower-educated people. Only on received support, there is less difference between respondents with high and low educational levels. Those with a medium level of education deviate less from lower educated respondents in terms of social capital. Institutional trust is the only factor on which respondents with a medium level of education return significantly higher scores than those with a low level of education. When it comes to income, we see that all other income groups return significantly higher scores on the four social capital indicators than the reference group of respondents who live on minimum wage. In general, there seems to be a linear positive correlation between income and social capital: the higher someone's income, the more social capital they have.

In Model 3 (Table A1, Appendix 1), we analyse the association between the various social capital indicators and the level of anxiety and stress experienced due to COVID-19. The results are fairly straightforward. All four indicators (social contacts, support received, general trust, and institutional trust) have a statistically significant negative correlation with anxiety and trust. The greater the respondents' social capital, the less anxiety and stress they experience. Only on the first indicator (social contacts), this correlation is considerably weaker. Apparently, merely having contacts with friends and family carries less weight when it comes to the mental health consequences of the COVID-19 crisis.

Finally, the full model with all variables is presented in Model 4 and Model 5 (Table 3). Model 5 contains only the significant correlations from Model 4 to create a more robust model. In our discussion of the results, we will focus only on Model 5. Firstly, we see that three of the four social capital indicators still have the expected negative impact on the level of anxiety and stress experienced as a result of the COVID-19 pandemic. People who receive support (or expect to) and have greater general and institutional trust experience relatively lower levels of anxiety and stress. These aspects of social capital offer some protection against the adverse mental health consequences of the COVID-19 crisis, although we should mention that the extra explained variance after including social capital into the model is rather limited (compare the *r*^2^ of Model 1 with Model 4 and 5). Only the last social capital indicator, i.e., the number of contacts with family and friends as such, is not significantly correlated with the experienced level of pandemic-related anxiety and stress, which is why it is not included in Model 5. Our outcomes differ from those of Makridis and Wu (2021) who found that only the trust dimension of social capital protects against the spread of the virus, rather than the social network dimension. These findings partly confirm both hypothesis 3 (only for received or expected informal support, not for social contacts as such) and hypothesis 4 of this study.

Secondly, we examine the mutual correlation between all three groups of factors in the analysis: does adding the factor of social capital to the analysis give us a better understanding of *why* some groups experience more anxiety and stress than others? Do young people, for example, experience more anxiety and stress partly because they have less protective social capital? When we compare the extent of the effects in Model 1 and Model 5 (Table 3), several salient facts emerge. Firstly, the gap between men and women in terms of experienced level of anxiety and stress widens slightly. Women not only experience more anxiety and stress than men, but the difference becomes greater when we realise that women generally have more (protective!) social capital. In other words: if women did not have more social capital than men, the difference in anxiety and stress compared to the men would be even greater.

The opposite is true for the difference between young people and older people, low and higher income groups, and between respondents with excellent and poor perceived health in both models. The general picture is that the differences between these categories shrink when we add the protective effect of social capital to the analysis, in Model 5. Young people, for example, experience more anxiety and stress as a result of the pandemic than older respondents, partly because they have less protective social capital than older people. We see the same when we compare the results for low-income and high-income respondents or respondents with poor or excellent perceived health. Low-income respondents and respondents with (very) poor perceived health experience more anxiety and stress than those with a higher income and those with better perceived health, partly because the two former categories of respondents have less protective social capital than the latter two. This was also the picture that Model 2 of Table A1 (see Appendix 1) showed.

We can conclude that the differences between men and women, between older and younger people, between people who are in good health and people whose health is not so good, and between high-income people and low-income people are partly the result of the fact that the former of each of these categories has more social capital that offers some level of protection against the adverse mental health consequences of the COVID-19 pandemic. To corroborate this finding, we finally examined whether the protective effect of social capital might work differently for the youngest age group (18–34 year olds) compared to older age groups. This turns out not to be the case (data not included in the table). The difference is that young people return lower scores on the various social capital indicators than older age groups.

## Conclusion and Discussion

In this article, we analysed the impact of the COVID-19 pandemic on the mental wellbeing of people in the Netherlands based on a large-scale and representative sample of the Dutch population. The data showed that the mental health consequences of the pandemic were considerable, especially in November 2020, on the eve of the predicted second wave of the pandemic. In November 2020, nearly 40% of respondents said they “feel they have nothing to look forward to”. A large number of respondents reported feeling more anxious or stressed since the COVID-19 pandemic broke out.

We found that the mental health consequences of the COVID-19 pandemic are not spread evenly over all groups of the Dutch population. Women, younger people, people on a minimum wage, and those in not-so-good or poor health experience significantly higher levels of anxiety and stress as a result of the COVID-19 crisis. Among men, older people, high-income groups, and people in good to excellent health, the mental health consequences of the pandemic are significantly less. However, our analysis did not show a statistically significant difference in mental health impact between people with and without a migration background. Initial differences in the extent to which respondents with and without a migration background experience anxiety and stress caused by the pandemic disappeared after controlling for differences in social status. When it comes to social status, we saw that low-income people experienced mental health consequences as a result of the COVID-19 pandemic more often than people from higher income groups, but we did not see this same difference when comparing people with lower and higher educational attainment. Financial uncertainty apparently has a greater effect on feelings of anxiety and stress caused by the COVID-19 pandemic than educational background does. In Bourdieu's terms, it is more about economic than cultural capital.

The central question of this study was, however, whether having social capital (defined both as being part of solidarity networks and as having trust in institutions and in other people) offers some protection against the adverse mental health impact of the pandemic. As expected, three of four social capital indicators used in the study, i.e., support received or expected, trust in institutions, and general trust, have a significant negative impact on experienced levels of anxiety and stress: the more support and trust, the lower the level of anxiety and stress. When people receive or expect support from others and/or when they have positive expectations of the trustworthiness of others and institutions such as governments, they experience less pandemic-related stress and anxiety. Only social contacts as such appeared not to correlate with less anxiety and stress. An explanation may be that having contact with people who are stressed themselves will also result in more anxiety and stress.

The scientific contribution of this study concerns on the one hand our finding that, in contrast to Ding et al. (2020) and Makridis and Wu (2021), both the network dimension and the trust dimension have an impact on perceived levels of anxiety and stress, and on the other hand that social capital has a different effect for different groups. If we take differences in the extent of social capital into account, the initial gap between men and women in terms of the level of anxiety and stress they experience becomes slightly larger. In other words: if women did not have more social capital than men, which they generally do, the difference in anxiety and stress compared to men would be even greater. The reverse applies when considering the differences in the level of anxiety and stress experienced within age, income, and health groups. If we include differences in social capital in the analysis, then the differences in the results within these groups become smaller. We can conclude that the differences between men and women, between older and younger people, between people who are in good health and those in poor health, and between high-income and low-income groups are *partly* the result of the fact that some groups have more social capital, which in one way or another offers protection against the adverse mental health consequences of the COVID-19 pandemic.

We are aware of the limitations of the study. Firstly, the cross-sectional study design limits the extent to which cause-effect relationships can be inferred from the findings. We analyse the relationships between social capital and expressed feelings of anxiety and stress, but we do not know the exact causal relationships. However, our research strategy is theoretically grounded in the scientific literature and we discuss our findings under the assumption that these are the correct causal directions. Secondly, our sociological approach has its limits. There is a long tradition of explaining (mental) health differences through differences in economic (income) or cultural capital (education) of social groups. More recently, the significance of social capital has been added to this literature, although including social capital in the models used in this study added only limited extra explained variance. Hence, social capital has a small but meaningful effect on relieving anxiety and stress in this study. We believe it is important to include insights from behavioural sciences and psychology about coping styles, resilience and personality factors in future studies on mental wellbeing in times of a pandemic. Thirdly, additional longitudinal research is important to gain closer insight into changes to social capital and how such changes can affect mental wellbeing. For some groups, for example, the social restrictions of the COVID-19 crisis may lead to a strongly reduced living environment, fewer social contacts, and less social support. Institutional trust may also decrease further among certain groups, which add to feelings that they are not less supported by important institutions. This, again, may result in increased feeling of anxiety and stress.

## Data Availability Statement

The raw data supporting the conclusions of this article will be made available by the corresponding author.

## Ethics Statement

The survey data for this research were collected by Kieskompas (“Election compass”), a Dutch political research organization that coordinates large research panels. Kieskompas asked informed consent from its panel members. The study is approved by the FSW Research Ethics Review Committee (RERC) of the Free University (VU), Amsterdam, The Netherlands.

## Author Contributions

ES wrote the initial text. JdB was responsible for all statistical analyses. GE and MvB finalized the article. All authors contributed to the article and approved the submitted version.

## Funding

This study was financed by the Netherlands Organisation for Health Research and Development (ZonMw), Project No. 10430032010034.

## Conflict of Interest

The authors declare that the research was conducted in the absence of any commercial or financial relationships that could be construed as a potential conflict of interest.

## Publisher's Note

All claims expressed in this article are solely those of the authors and do not necessarily represent those of their affiliated organizations, or those of the publisher, the editors and the reviewers. Any product that may be evaluated in this article, or claim that may be made by its manufacturer, is not guaranteed or endorsed by the publisher.

## Footnotes

^1^See also data published by Statistics Netherlands: https://opendata.cbs.nl/#/CBS/nl/dataset/83780NED/table?ts=1590569035685.

^2^We used Principal Component Analysis (PCA) to see if all relevant items could be reduced into one factor (social capital). The PCA test showed that we are unable to summarize all items into one single factor, because not all items load on the same component. Additionally, one single component does not (sufficiently) capture the variance between the items. Furthermore, Confirmatory Factor Analysis (CFA) was performed to test the validity of the subscales. The results show that not all fit indices indicate a good model fit for each sub scale separately (see Table A2 in Appendix 2, cf. Beaujean, 2014). A second-order CFA model was also tested to see if a single factor (social capital) could explain the covariances between each sub scale. However, not all fit indices indicate good fit (possibly because some of the subscales themselves were only moderately reliable). All in all, we argue that according to the literature and the “moderate” fit of the separate subscales it is informative to use them as an estimation of the related constructs.

^3^According to Field (2009, p. 224), a VIF > 10 is seen as problematic in terms of multi-collinearity. All of our VIF values are below this criterion.

^4^Weighting may also be a factor here. Bivariate analyses were conducted on the weighted dataset for this study, while the multivariate regression analyses were conducted on the unweighted dataset. But also in the unweighted dataset, it still turns out that non-Western migrants experience significantly more (*p* < *0.05*) stress than native Dutch people.

## Appendix 1

**Table A1:** Determinants of social contacts, support received, trust in people in general, trust in institutions and anxiety and stress resulting from COVID-19 (Linear regression).

	**Model 2a contact**				**Model 2b support received**				**Model 2c trust in people**				**Model 2d institutional trust**				**Model 3 anxiety and stress**			
	**B**		**SE B**	**ß**	**B**		**SE B**	**ß**	**B**		**SE B**	**ß**	**B**		**SE B**	**ß**	**B**		**SE B**	**ß**
(Constante)	1,987	^***^	0,056		1,922	^***^	0,026		2,300	^***^	0,046		2,955	^***^	0,049					
Gender (male = ref)																				
Female	0,163	^***^	0,015	0,080	0,062	^***^	0,007	0,066	0,129	^***^	0,012	0,075	0,010		0,013	0,006				
Age (in years)	0,005	^***^	0,000	0,087	0,005	^***^	0,000	0,171	0,008	^***^	0,000	0,146	−0,001	^**^	0,000	−0,026				
Migration background (non = ref)																				
Western	0,020		0,024	0,006	−0,011		0,011	−0,007	−0,052	^**^	0,019	−0,019	−0,057	^**^	0,021	−0,019				
Non-Western	−0,130	^**^	0,043	−0,022	−0,019		0,020	−0,007	−0,162	^***^	0,035	−0,032	−0,070		0,037	−0,013				
Living situation (single = ref)																				
More persons household	0,076	^***^	0,018	0,033	0,010		0,008	0,010	0,058	^***^	0,015	0,030	−0,040	^*^	0,016	−0,020				
Health ((very) bad = ref)																				
Moderate	0,151	^***^	0,041	0,057	0,024		0,019	0,019	0,152	^***^	0,034	0,068	0,243	^***^	0,036	0,103				
Good	0,246	^***^	0,038	0,121	0,075	^***^	0,018	0,080	0,371	^***^	0,032	0,216	0,425	^***^	0,034	0,235				
Very good	0,322	^***^	0,041	0,127	0,126	^***^	0,019	0,108	0,468	^***^	0,034	0,218	0,500	^***^	0,036	0,221				
Education (lower = ref)																				
Secondary	0,049		0,027	0,022	0,030	^*^	0,012	0,030	0,055	^*^	0,022	0,030	0,122	^***^	0,023	0,063				
Higher	0,118	^***^	0,026	0,058	0,025	^*^	0,012	0,027	0,186	^***^	0,022	0,108	0,304	^***^	0,023	0,168				
Income (Minimum = ref)																				
Minimum to modal	0,147	^***^	0,031	0,055	0,050	^***^	0,014	0,041	0,079	^**^	0,025	0,035	0,079	^**^	0,027	0,033				
Modal to 2x modal	0,148	^***^	0,028	0,068	0,064	^***^	0,013	0,065	0,121	^***^	0,023	0,066	0,163	^***^	0,025	0,085				
2x modal to 3x modal	0,209	^***^	0,030	0,092	0,084	^***^	0,014	0,080	0,196	^***^	0,025	0,103	0,229	^***^	0,026	0,114				
More than 3x modal	0,230	^***^	0,032	0,088	0,082	^***^	0,015	0,068	0,259	^***^	0,026	0,118	0,320	^***^	0,028	0,138				
Social contacts (0-5)																	−0,014	^*^	0,006	−0,016
Support received (1-4)																	−0,197	^***^	0,014	−0,102
Trust in people in general (1-5)																	−0,115	^***^	0,008	−0,108
Trust in institutions (1-5)																	−0,079	^***^	0,007	−0,079
	F = 36,97^***^; R^2^ = 0.026; adj. R^2^ = 0.025				F = 52,27^***^; R^2^ = 0.036; adj. R^2^ = 0.036				F = 96,52^***^; R^2^ = 0.065; adj. R^2^ = 0.065				F = 87,29^***^; R^2^ = 0.059; adj. R^2^ = 0.059				F = 235,69^***^; R^2^ = 0.042; adj. R^2^ = 0.042			

*B, unstandardized regression coefficient; SE B, standard error of the regression coefficient; ß, standardized regression coefficient; R^2^, explained variance; adj. R^2^, adjusted explained variance*;

* *p < 0.05*,

** *p < 0.01*,

*** *p < 0.001 (two tailed)*.

## Appendix 2

**Table A2:** Fit-indices of the confirmatory factor analyses: three sub scales separately and one higher-order model.

**Model specification**	**X^2^**	***df***	**CFI**	**SRMR**	**RMSEA**
**Social contacts** 4 items	2389***	2	0.875	0.072	0.22
**Social support** 4 items	756.45***	2	0.944	0.035	0.124
**Institutional trust** 4 items	1619.39***	2	0.893	0.045	0.18
**Social capital** (Second-order-model for the three sub scales)	5785.85***	51	0.878	0.041	0.069

*A non-significant X^2^, CFI> 0.90, SRMR < 0.08, and RMSEA < 0.06 indicate a good model fit (Beaujean, 2014)*.
